# Efficiency of induction of Shiga-toxin lambdoid prophages in *Escherichia coli* due to oxidative and antibiotic stress depends on the combination of prophage and the bacterial strain

**DOI:** 10.1007/s13353-019-00525-8

**Published:** 2019-12-06

**Authors:** Michalina Filipiak, Joanna M. Łoś, Marcin Łoś

**Affiliations:** 1 Department of Bacterial Molecular Genetics, Faculty of Biology, University of Gdansk, Wita Stwosza Street 59, 80-308 Gdansk, Poland; 2 Phage Consultants, Partyzantow Street 10/18, 80-254 Gdansk, Poland

**Keywords:** Prophage, Oxidative stress, Antibiotic stress, Shiga toxin, SOS response, Prophage induction

## Abstract

In the study presented here, we tested, how large a fraction of lysogenic culture was undergoing filamentation, which could indicate triggering of the SOS response or SOS-independent prophage induction that is also known to cause cell filamentation. Here, antibiotic stress was triggered by adding mitomycin C and oxidative stress was induced by hydrogen peroxide. Observation of bacterial cells under an optical microscope revealed more filamenting cells for lysogenic *Escherichia coli* than for strains not carrying a prophage. Moreover, the amount of filamenting cells depended not only on the stress agents used and the type of the prophage, but also on the host. During induction of the 933W prophage, the resulting phage titer and the amount of elongating cells were different when using *E. coli* O157:H7 EDL933 clinical isolate and the *E. coli* MG1655 laboratory strain. The amount of filamenting cells correlates well with the observed phage titers.

## Introduction

Enterohemorrhagic *Escherichia coli* (EHEC) is a highly pathogenic bacterial strain responsible for bloody diarrhea and hemorrhagic colitis. Prolonged infection can develop into a hemolytic-uremic syndrome (HUS) in approximately 15% of young patients diagnosed with EHEC O157:H7 infection (Karpman and Ståhl 2014; Karch et al. 2005). The main virulence factor is the Shiga toxin, encoded by the *stx1* and *stx2* genes, which are located among the late genes of lambdoid prophages. Since the *stx* genes are effectively expressed after prophage induction and during subsequent lytic development of the phage, studies on bacterial response to different stress conditions may help to understand the pathogenicity of EHEC.

Prophage induction has been reported to occur under many stress conditions, such as oxidative stress, osmotic stress, and antibiotic stress. Conditions where the bacterial SOS response is activated lead to prophage induction in case of many phage groups, including lambdoid prophages. In the model phage from this group—phage λ, activation of the RecA protein leads to cleavage of the prophage repressor that no longer inhibits prophage promoters (Roberts et al. 1978). However, not only antibiotics that directly damage DNA or proteins that take part in DNA metabolism can induce prophages, inhibition of host termination or growth phase changes also cause prophage induction (Ramirez et al. 1999; Menouni et al. 2013; Węgrzyn and Węgrzyn 2005). Moreover, Shiga toxin production depends not only on an antibiotic used, but also on a given strain (Grif et al. 1998). Different classes of antibiotics have been tested for their influence on the Shiga toxin production by *E. coli* O157:H7 EDL933 strain. Streptomycin, sulfamethoxazole, and ciprofloxacin strongly increase the *stx*2 gene expression (McGannon et al. 2010; Grif et al. 1998).

Another antibiotic which is commonly used in the laboratory as a prophage-inducing agent is mitomycin C, which has also antitumor properties. A standard mitomycin C concentration that kills 50% of *E. coli* K-12 cells is 1.4 μg/ml, but in *E. coli* K-12(λ), it is only 0.5 μg/ml, due to the prophage induction (Muschel and Schmoker 1966). In most studies, a concentration of 1 μg/ml is used. However, to achieve increased Stx2 production by O157:H7 EDL933, a concentration of 2 μg/ml was deemed as the most effective (Tyler et al. 2013). Mitomycin C acts on DNA in three possible ways: promoting (a) cross-linking events, (b) alkylation and not necessarily cross-linking, and (c) strand breakage. Degradation of DNA observed to accompany the cytotoxic action of mitomycin C is largely due to release of the O_2_ free radical. However, mitomycin C does not inactivate the cellular protective enzymes, such as superoxide dismutase or catalase (Lown et al. 1976). It has been proven that mitomycin C induces synthesis of the *Salmonella* toxin, *Cholera* toxin, and Shiga toxin in various strains (Yee et al. 1993; Houston et al. 1981; Molina and Peterson 1980). Apart from toxin induction, mitomycin C causes cell lysis that has been correlated with bacteriophage induction (Gemski et al. 1978). Our previous study has shown that mitomycin C and hydrogen peroxide induce the Shiga toxin-encoding prophages: 22Δtox, 27Δtox, 32Δtox, ϕ24_B_, and 933ΔWtox, from an *E. coli* MG1655 strain (Łoś et al. 2009) and the ST2-8624 prophage from the O157:H7 strain (Łoś et al. 2010).

Depending on the H_2_O_2_ concentration, killing of *E. coli* cells can proceed in two modes. The first one occurs in the range of 1 to 3 mM H_2_O_2_ due to DNA damage and requires active metabolism of the cell during exposure. The second mode is due to general damage and exhibits a dose-dependent increase in damage, up to at least 50 mM of H_2_O_2_. There is also a suggestion that hydrogen peroxide activates the SOS response which protects bacteria from the first mode of killing by enhancing DNA repair. Low concentrations of H_2_O_2_ also promote phage DNA replication in *Lactococcus lactis* cultures (Ho et al. 2016).

Another phenomenon is filamentation of the bacterial cell due to induction of the SOS system, which could be triggered by many different conditions resulting in appearance of single-stranded DNA fragments in the cell (Baharoglu and Mazel 2014). This manifests a mechanism which prevents bacteria from dividing until DNA damage is repaired (Imlay and Linn 1987). Filamentation can be used as an indicator of the SOS response under stress conditions and can be easily observed under an optical microscope. Also, it has been reported that 0.5-μg/ml concentration of mitomycin C promotes elongation of Chinese hamster ovary cells (CHO) infected with *Salmonella* during release of the *Salmonella* toxin (Molina and Peterson 1980). It was also reported that tif-1 mutation (Castellazzi et al. 1972) in the *recA* gene causes cell filamentation at elevated temperatures by direct activation of the RecA protein, without causing DNA damage (McEntee and Weinstock 1981). Filamentation may also be the result of induction of the lambda prophage and the activity of phage-encoded *kil* gene whose product, by interacting with FtsZ and ZipA, causes cell filamentation due to block of FtsZ assembly (Haeusser et al. 2014). The *kil* gene seems to be quite widespread between different lambdoid prophages, including the Shiga toxin bearing representatives of this phage group, such as, e.g., 933W (Plunkett III et al. Plunkett et al. 1999) and ϕ24B (Smith et al. 2012). Thus, the increase of the length of bacterial cells caused by filamentation seems to be a relatively good indicator of the prophage induction, regardless of the exact causative agent of the induction itself, even in the absence of RecA activation.

Our previous study has shown different efficiency of induction of the prophages tested (Łoś et al. 2009). In this study, we wanted to assess, on the basis of cell filamentation, how large a fraction of cells in the bacterial population contains induced prophages after treatment with mitomycin C and hydrogen peroxide. We also investigated if the presence of a prophage can influence sensitivity of bacteria to oxidative and antibiotic stressors. We hoped that the obtained results will help to explain the previously observed differences in efficiency of prophage induction.

## Materials and methods

### Bacterial strains

*E. coli* strains used in this study are listed in Table 1.

**Table 1:** Bacterial strains

*E. coli* strain	Source or reference
C600	Our collection
MG1655	ATCC 47076
O157:H7 EDL933	Our collection
MG1655(933WΔtox)	Our collection
MG1655(22Δtox)	Our collection
MG1655(27Δtox)	Our collection
MG1655(32Δtox)	Our collection
MG1655(ϕ24_B_)	Our collection
MG1655(λpapa)	Our collection

### Prophage induction

For each experiment, conducted in triplicate, *E. coli* cells lysogenized with one of the tested phages were grown overnight in LB medium (Roth) at 37 °C, with 130 rpm shaking (Grant OLS200). The next day, the culture was diluted 1:100 in a fresh LB medium (Roth) and grown until OD_600_ = 0.1~0.15. At that point, the culture was divided into three aliquots, where one was left as a control without any inducing agent (to enable observation of only spontaneous induction of the prophage), another one was supplemented with mitomycin C (Sigma-Aldrich) to the final concentration of 1 μg/ml, and the third one was supplemented with 3 mM H_2_O_2_ (Sigma-Aldrich). The concentrations of inducers were chosen on the basis of our previous studies. The concentration of 3 mM H_2_O_2_ and 1 μg/ml was shown to be optimal for induction of prophages in *E. coli* MG1655 (Łoś et al. 2009, 2010). Cultures were grown with shaking for 4 h. Every hour or every 30 min, depending on the strain, each culture was assayed for optical density at 600 nm (Eppendorf BioPhotometer 6131), phage titration, and enumeration of elongated cells under optical microscopy (Zeiss Primo Star) Table 2.

**Table 2:** Phage titer and % of filamenting cell in untreated culture, after adding 1 μg/ml mitomycin C and 3 mM H_2_O_2_. ND not detected, *p* value for *t* test: ***p < 0.01, **p < 0.05, *p < 0.1

Bacterial strain	Inducing agent	Time [min]															
		30		60		90		120		150		180		210		240	
		Filamentation [%]	Phage titer	Filamentation [%]	Phage titer	Filamentation [%]	Phage titer	Filamentation [%]	Phage titer	Filamentation [%]	Phage titer	Filamentation [%]	Phage titer	Filamentation [%]	Phage titer	Filamentation [%]	Phage titer
MG1655	No	ND	ND	1.45 ± 0.50	ND	ND	ND	1.54 ± 0.07	ND	ND	ND	1.56 ± 0.50	ND	ND	ND	2.03 ± 0.34	ND
	Mitomycin C	ND	ND	31.25 ± 2.39 ***	ND	ND	ND	76.79 ± 7.39 ***	ND	ND	ND	69.57 ± 6.27 ***	ND	ND	ND	67.74 ± 5.15 ***	ND
	H_2_O_2_	ND	ND	12.86 ± 2.51 ***	ND	ND	ND	31.51 ± 3.22 ***	ND	ND	ND	21.11 ± 1.44 ***	ND	ND	ND	11.36 ± .41 ***	ND
MG1655 (λpapa)	No	1.56 ± 0.77	1.37*10^5^ ± 1.29*10^5^	1.18 ± 0.22	4.26*10^5^ ± 2.81*10^5^	1.69 ± 0.39	1.29*10^5^ ± 6.73*10^4^	1.03 ± 0.21	7.11*10^5^ ± 1.97*10^5^	2.29 ± 1.34	1.15*10^6^ ± 7.40*10^5^	1.12 ± 0.13	1.71*10^6^ ± 1.70*10^6^	1.44 ± 0.68	4.37*10^5^ ± 3.26*10^5^	1.16 ± 0.12	7.05*10^5^ ± 1.41*10^5^
	Mitomycin C	69.93 ± 6.81 ***	2.42*10^5^ ± 3.49*10^5^	78.13 ± 6.65 ***	3.41*10^5^ ± 4.02*10^5^	66.68 ± 6.29 ***	4.78*10^6^ ± 6.34*10^6^	85.56 ± 5.09 ***	6.80*10^7^ ± 2.50*10^7^	81.48 ± 3.21 ***	3.59*10^8^ ± 4.17*10^8^	76.85 ± 5.78 ***	2.28*10^8^ ± 3.24*10^8^	70.46 ± 6.01 ***	6.00*10^8^ ± 3.82*10^8^	81.67 ± 2.36 ***	4.24*10^8^ ± 4.75*10^8^
	H_2_O_2_	7.43 ± 7.12 *	4.12*10^4^ ± 5.16*10^4^	35.5 ± 21.75 **	5.04*10^4^ ± 2.85*10^4^	30.1 ± 17.63 **	8.12*10^6^ ± 7.02*10^6^	18.57 ± 3.78 ***	2.15*10^7^ ± 1.61*10^7^	9.26 ± 5.16 **	5.08*10^7^ ± 6.01*10^7^	10.37 ± 6.12 **	4.24*10^7^ ± 4.34*10^7^	12.9 ± 10.92 *	8.27*10^6^ ± 2.41*10^6^	7.25 ± 6.72 *	6.80*10^6^ ± 5.66*10^5^
MG1655 (22Δtox)	No	ND	ND	3.44 ± 1.50	2.19*10^5^ ± 1.92*10^5^	ND	ND	1.33 ± 0.58	6.27*10^5^ ± 2.05*10^5^	ND	ND	1.16 ± 0.44	1.72*10^6^ ±9.46*10^5^	ND	ND	1.45 ± 0.21	2.24*10^7^ ± 1.89*10^7^
	Mitomycin C	ND	ND	75.24 ± 7.52 ***	4.23*10^5^ ± 4.65*10^5^	ND	ND	83.49 ± 1.81 ***	5.08*10^6^ ± 6.08*10^6^	ND	ND	87.22 ± 6.31 ***	1.56*10^9^ ± 2.23*10^8^	ND	ND	86.11 ± 12.73 ***	5.47*10^10^ ± 2.72*10^10^
	H_2_O_2_	ND	ND	28.53 ± 5.42 ***	2.47*10^5^ ± 3.06*10^5^	ND	ND	36.00 ± 9.24 ***	6.91*10^6^ ± 4.69*10^6^	ND	ND	15.28 ± 3.38 ***	1.11*10^8^ ± 4.39*10^7^	ND	ND	8.08 ± 4.87 **	6.00*10^8^ ± 3.12*10^8^
MG1655 (27Δtox)	No	ND	ND	2.26 ± 0.88	2.80*10^5^ ± 3.19*10^5^	ND	ND	2.13 ± 1.87	6.12*10^5^ ± 6.22*10^5^	ND	ND	1.34 ± 0.29	4.6*10^6^ ± 3.3*10^6^	ND	ND	2.03 ± 0.85	5.87*10^7^ ± 6.43*10^7^
	Mitomycin C	ND	ND	73.97 ± 3.57 ***	4.57*10^5^ ± 3.65*10^5^	ND	ND	82.80 ± 9.96 ***	1.51*10^7^ ± 2.17*10^7^	ND	ND	82.03 ± 7.15 ***	1.19*10^10^ ± 5.10*10^9^	ND	ND	80.00 ± 0.00 ***	7.87*10^10^ ± 4.16*10^10^
	H_2_O_2_	ND	ND	6.74 ± 1.10 ***	1.25*10^5^ ± 4.69*10^4^	ND	ND	66.3 ± 11.28 ***	5.52*10^6^ ± 7.69*10^6^	ND	ND	12.86 ± 2.47 ***	2.71*10^8^ ± 2.23*10^8^	ND	ND	10.32 ± 3.82 **	5.20*10^8^ ± 8.00*10^7^
MG1655 (32Δtox)	No	ND	ND	1.70 ± 0.63	5.60*10^4^ ± 2.43*10^4^	ND	ND	1.44 ± 0.49	3.32*10^5^ ± 3.36*10^5^	ND	ND	0.99 ± 0.44	2.53*10^5^ ± 3.01*10^5^	ND	ND	1.18 ± 0.22	9.65*10^5^ ± 7.24*10^5^
	Mitomycin C	ND	ND	80.02 ± 1.46 ***	3.60*10^4^ ± 6.93*10^3^	ND	ND	85.84 ± 4.09 ***	2.79*10^6^ ± 2.44*10^6^	ND	ND	89.33 ± 5.13 ***	7.24*10^8^ ± 5.75*10^8^	ND	ND	80.06 ± 8.10 ***	9.84*10^9^ ± 8.24*10^9^
	H_2_O_2_	ND	ND	70.24 ± 6.53 ***	5.20*10^3^ ± 4.18*10^3^	ND	ND	21.20 ± 8.24 ***	1.12*10^6^ ± 1.80*10^6^	ND	ND	12.65 ± 6.03 ***	1.56*10^7^ ± 1.16*10^7^	ND	ND	10.24 ± 4.76 ***	5.40*10^7^ ± 4.33*10^7^
MG1655 (ϕ24B)	No	ND	ND	1.41 ± 0.44	1.32*10^5^ ± 1.74*10^4^	ND	ND	1.44 ± 0.51	1.79*10^5^ ± 1.63*10^5^	ND	ND	1.03 ± 0.38	9.47*10^5^ ±1.01*10^5^	ND	ND	1.60 ± 0.54	4.07*10^6^ ± 2.52*10^6^
	Mitomycin C	ND	ND	72.22 ± 4.81 ***	2.75*10^5^ ± 2.49*10^5^	ND	ND	84.11 ± 0.84 ***	8.96*10^6^ ± 5.00*10^6^	ND	ND	84.95 ± 1.67 ***	1.31*10^9^ ± 6.89*10^8^	ND	ND	77.47 ± 8.47 ***	9.07*10^9^ ± 5.08*10^9^
	H_2_O_2_	ND	ND	9.23 ± 1.14 ***	1.19*10^5^ 8.33*10^3^	ND	ND	35.35 ± 2.27 ***	9.87*10^5^ ± 4.60*10^5^	ND	ND	22.14 ± 1.04 ***	1.15*10^8^ ± 3.23*10^7^	ND	ND	12.51 ± 0.98 ***	1.36*10^8^ ± 4.00*10^6^
MG1655 (933WΔtox)	No	ND	ND	2.22 ± 1.58	5.87*10^4^ ± 1.80*10^4^	ND	ND	2.67 ± 0.88	5.19*10^5^ ± 3.49*10^5^	ND	ND	1.67 ± 0.58	2.27*10^5^ ± 1.89*10^5^	ND	ND	1.69 ± 0.04	1.74*10^6^ ± 2*10^6^
	Mitomycin C	ND	ND	80.49 ± 2.64 ***	1.55*10^5^ ± 1.43*10^5^	ND	ND	88.87 ± 2.77 ***	1.47*10^6^ ± 7.11*10^5^	ND	ND	83.0 ± 10.24 ***	6.2*10^8^ ± 3.89*10^8^	ND	ND	80.61 ± 1.05 ***	1.08*10^10^ ± 9.03*10^9^
	H_2_O_2_	ND	ND	20.0 ± 12.67 **	1.19*10^6^ ± 2.02*10^6^	ND	ND	31.96 ± 9.72 **	8.24*10^6^ ± 1.19*10^7^	ND	ND	14.10 ± 6.28 ***	2.99*10^8^ ± 2.96*10^8^	ND	ND	9.51 ± 4.23 **	9.92*10^8^ ± 1.57*10^9^
EDL933	No	4.41 ± 0.11	1.00 ± 0.00	4.67 ± 1.80	2.02*10^1^ ± 1.80* 10^1^	2.84 ± 0.58	5.60*10^2^ ± 3.80*10^2^	3.57 ± 0.38	1.20*10^3^ ± 8.60*10^2^	2.67 ± 0.21	2.82*10^3^ ± 2.27*10^3^	3.87 ± 0.38	3.34*10^3^ ± 2.59*10^3^	3.68 ± 0.96	3.64*10^3^ ± 2.32*10^3^	4.80 ± 2.64	4.24*10^3^ ± 2.34*10^3^
	Mitomycin C	76.25 ± 9.67 ***	1.00 ± 0.00	75.56 ± 18.18 ***	3.62*10^1^ ± 2.20 *10^1^	84.2 ± 11.88 ***	1.28*10^4^ ± 1.16 *10^4^	71.91 ± 7.12 ***	9.52*10^4^ ± 8.56*10^4^	60.00 ± 20.46 ***	4.54*10^5^ ± 2.53*10^5^	77.27 ± 14.18 ***	5.31*10^5^ ± 3.24*10^5^	74.29 ± 3.85 ***	5.88*10^5^ ± 3.48*10^5^	69.57 ± 11.02 ***	1.06*10^6^ ± 5.89*10^5^
	H_2_O_2_	37.1 ± 17.82 **	1.00 ± 0.00	42.59 ± 12.73 ***	1.64*10^1^ ± 2.40*10^1^	38.6 ± 10.51 ***	2.20*10^2^ ± 5.70*10^3^	33.33 ± 6.84 ***	1.18*10^4^ ± 4.98*10^4^	31.82 ± 9.14 ***	1.48*10^5^ ± 2.44*10^5^	25.52 ± 0.90 ***	3.85*10^5^ ± 2.27*10^5^	22.02 ± 2.19 ***	2.96*10^5^ ± 1.89*10^55^	30.68 ± 2.41 ***	4.74*10^5^ ± 5.75*10^5^

### Phage titration procedure

For titration of 22Δtox, 27Δtox, 32Δtox, ϕ24B, and 933WΔtox phages, bottom agar was supplemented with 2.5 μg/ml of chloramphenicol, according to Łoś et al. (2008). For the 933W phage, full-plate titration was used and the bottom agar was supplemented with 1.5 μg/ml of tetracycline. In both cases, top agar was supplemented with 10 mM CaCl_2_ and 10 mM MgCl_2_. Plates were incubated overnight at 37 °C.

### Optical microscopy and enumeration of cells

To determine if bacterial cells are elongated, the overall length of cells was measured. The length of the cells under all conditions tested was calculated according to the average length of the cells grown under control conditions. Cells longer than 5 μm were considered as elongated at the start of the experiment, before either of the inducing agents were used. However, during the progress of experiments with mitomycin C, elongated cells were considered as those that exceeded 10 μm (after 30 min or 1 h, depending on the tested strain), then 15 μm after 2 h, and reaching the maximum of 50 μm at the end of the experiment. In case of treatment with hydrogen peroxide, elongated cells are easily distinguished from the surrounding cells and those longer than 12 μm after 2 h of treatment were scored as elongated; their length was reaching a maximum of 30 μm after 4 h. Under control conditions, cells that were longer than 5 μm were considered as elongated during the course of experiment. Measurements of length of the bacterial cells were done based on pictures taken with AxioCamERc5s of the Zeiss Primo Star microscope, using 100× magnifying lens. The number of bacterial cells was counted in the Neubauer chamber, using 10 μl of bacterial culture.

## Results

**Fig. 1.**
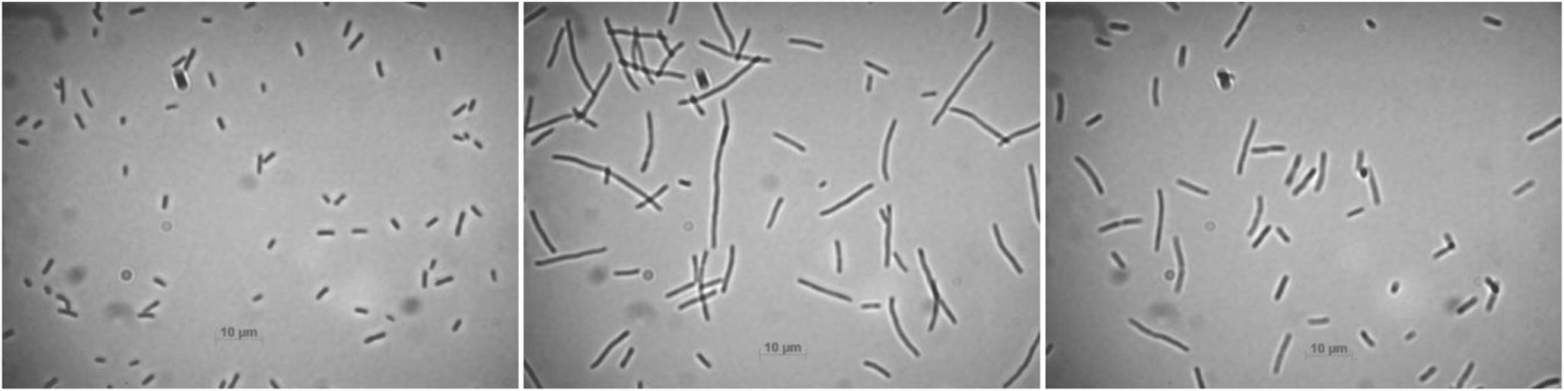
Optical microscopy for MG1655(22Δtox) at 1 hour h after adding inducing agent. Left panel untreated cell, middle panel with 1 μg/ml mitomycin C, and right panel with 3 mM H_2_O_2_.

Cell filamentation occurs during the SOS response, but it can be a result of induction of prophage itself without triggering the SOS regulon. For example, the *kil* gene of phage lambda, when expressed, causes cell filamentation as well (Haeusser et al. 2014), which can be easily observed using optical microscopy. We used this method for counting the percentage of cells responding to stress conditions. The standard size of *E. coli* cell is reported by many authors to range from 2 μm in length and 0.8 μm in diameter (Pierucci 1978) to 3.3 μm in length (Gangan and Athale 2017); however, these values can differ depending on growth phase and growth conditions (Arends and Weiss 2004). During the course of our experiment, the length of untreated, control bacteria was different than what is described in the literature. Untreated bacteria, grown under control conditions, tended to produce shorter cells over time, as the growth phase was progressing from exponential towards stationary, whereas different stress agents promoted development of bacteria in modified shapes, mostly elongated or filamentous, often in the form of three to four connected cells. Under control conditions, without any inducing agents added, most of the cells were rod-shaped, and at the end of the experiments, the cells became more round and smaller, which is associated with entering the stationary phase. In experiments utilizing mitomycin C, the cells lengthened to approximately 20 μm in the first hour and elongated even more in the next hours. In cultures undergoing exposure to hydrogen peroxide, in most cases, the cells were elongated as well. However, this happened for a shorter period of time when compared to exposure to mitomycin C. Typical example of cell filamentation observed during experiments is shown on Fig. 1.

**Fig. 2.**
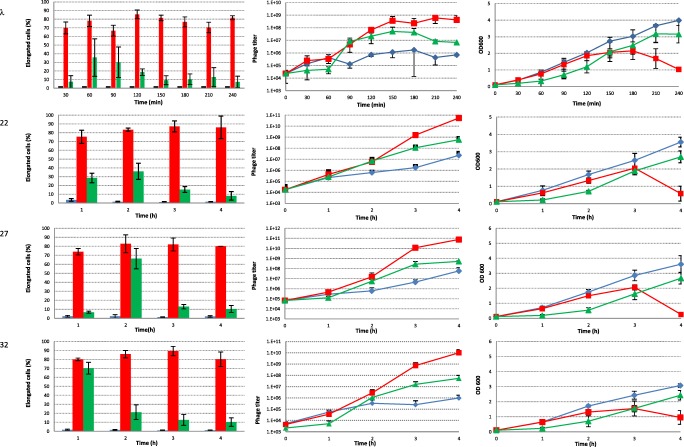

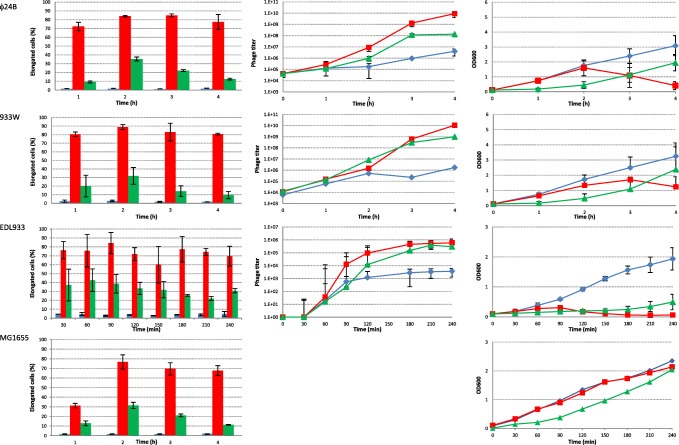
Number of elongated cells, phage titer, and OD600 measurements for all tested strains. Left panel shows the number of elongated cells in the control experiment (blue bars), after addition of 1 μg/ml mitomicin C (red bars) and 3 mM H_2_O_2_ (green bars). The middle panel shows the phage titer for control experiment (blue diamonds), after addition of 1 μg/ml mitomycin C (red squares), and 3 mM H_2_O_2_ (green triangles). Right panel shows OD600 measurements for the control experiment (blue diamonds), after addition of 1 μg/ml mitomycin C (red squares) and 3 mM H_2_O_2_ (green triangles)

In all cases, growth of the cultures without any inducing agents added was accompanied by spontaneous induction of prophages. Results from cell filamentation and phage titer for each strain are presented in Fig. 2 and numerical data with statistics is presented in Table 1. The amount of phage released in the events of spontaneous induction was strictly dependent on the prophage type and reached maximum values in the range of 2 × 10^6^/ml in the case of the lambda phage, and up to 6 × 10^7^ in the case of phage 27 (Fig. 2, middle panel). The titer of phage 933W induced from the EDL933 strain followed this general trend, but due to the fact that the efficiency of plating of this phage is much lower, it cannot be directly compared in terms of absolute numbers. The phage titration method (Łoś et al. 2008) used for the 933WΔtox phage, employing subinhibitory concentrations of antibiotics in both layers of agar, turned out to be inefficient for phage 933W. Even though a previously described method for titration of the Stx2 phage of *E. coli* O157:H7 strain (Islam et al. 2012) was used with a modification which was shown in another report to be the most effective (Łoś et al. 2008), no visible plaques were observed on plates. Eventually, 1.5 μg/ml concentration of tetracycline in the bottom agar, addition of Mg^2+^ and Ca^2+^ ions to the top agar, and full plate titration was the most successful way of obtaining plaques. Spontaneous induction was observed, but the amount of cells which underwent elongation was small. Spontaneous induction of prophages in various bacteria is often caused by SOS activation in a small fraction of cells in a culture, which is not exposed to any DNA-damaging chemicals; however, in some cases, prophages can be induced without triggering the SOS response, and it may even result from stochasticity in gene expression, leading to a critically low level of the prophage repressor (Nanda et al. 2014; Nanda et al. 2015). Despite real cause of prophage induction, in our experimental setting, this induction should result in cell filamentation due to either SOS induction or blocking of FtsZ polymerization by Kil (Haeusser et al. 2014).

To verify to what extent elongation of bacterial cells is promoted by the action of mitomycin C or H_2_O_2_ alone, control experiments were conducted with *E. coli* strain MG1655. Our observations under microscope indicated that cells were elongated in response to mitomycin C, reaching the highest amount in the second hour of growth. In the culture treated with H_2_O_2_, the percentage of filamenting cells was much lower when compared to cells treated with mitomycin C. However, similarly to experiments with mitomycin C, the highest amount of filamentation was reached in the second hour of experiments. At the end of the experiment, decrease in the amount of filamenting cells over time was much faster for hydrogen peroxide than for mitomycin C treatment. Under control conditions, only up to 2.5% of cells were elongated for the whole duration of experiment (Fig. 2, left panel).

In the cultures with hydrogen peroxide added, the released phages reached three orders of magnitude higher numbers than the phages originating from spontaneous induction events. As filamentation itself will be an indicator of either SOS regulon induction or induction of phage itself due to Kil protein action (Haeusser et al. 2014), the observed effect of phage titer increase seems to be due to increased frequency of induction, not the increased productivity of filamenting cells, as all or at least vast majority of cells with induced prophage should undergo elongation. Filamentation caused by addition of hydrogen peroxide followed a pattern which was dependent on the prophage used. Usually, the increase in the amount of filamenting cells was sharp and rather transient, followed by a decrease in the elongated cell fraction. The size of the elongated cell fraction was different, and some patterns could be distinguished. For the MG1655(ϕ24_B_) strain, the amount of filamenting cells was similar to the one observed for the MG1655 control strain, throughout the whole experiment. In the case of the 22Δtox and 933WΔtox prophages, the amount of elongated cells in the first hour was twice as high as for the control strain without a prophage, and in the next hours of experiment, it was similar to the values observed for the MG1655 control strain. Presence of the lambda prophage had increased the amount of filamenting cells by about 3-fold in the first hour of bacterial growth under conditions of oxidative stress, when compared to the MG1655 strain. In the second hour of experiment, this amount was already lower than for the control strain. A similar pattern was seen for the 32Δtox prophage, but the difference in amount of elongated cells was 6-fold higher than for the control strain in the first hour. In the case of the 27 and 32 prophages, the amount of elongated cells constituted a majority of the culture, but it was followed by a rapid decrease in the amount of this fraction. The maximum increase in the share of elongated cells in the culture was visible in the case of prophage 27 in the second hour, and in the case of prophage 32 in the first hour after addition of hydrogen peroxide. In the case of other prophages carried by the MG1655 strain, the increase in elongation was not so sharp, and it was accompanied by a more gradual disappearance of elongated cells from the culture. The maximum fraction of elongated cells was visible at 2 h post-addition of hydrogen peroxide (Fig. 1, left panel). For a majority of prophages, the disappearance of elongated cells was correlated with the fastest increase in phage titer in the culture (Fig. 1, middle panel), which may suggest a correlation between their disappearance and phage-invoked lysis.

The results presented here indicate that sensitivity of cells to oxidative stress depends not only on the type of prophage but also on the bacterial strain itself. A good example is the O157:H7 EDL933 strain, which after induction with hydrogen peroxide maintained a high level of elongated cells for 4 h of the experiment (Fig. 2, left panel), and at the same time had released a high amount of phage particles. Although not observed in the MG1655 background, the 933W hydrogen peroxide induction in its original strain EDL933 background produced a titer of free phages comparable with that obtained by induction with mitomycin C. After induction, the EDL933 strain also produced the 933W phage much faster when compared to MG1655 (Fig. 2, middle panel).

Mitomycin C addition was used to compare the effectiveness of prophage induction and induction of filamentation by hydrogen peroxide. In all cases, mitomycin C was more effective than hydrogen peroxide, and in all cases, its addition caused lysis of the bacterial culture, which started approximately 3 h after addition of this compound when a prophage was present in the strain (Fig. 1, right panel). During the course of the experiment, starting 1 h post-induction, the fraction of elongated cells in mitomycin C-treated cultures was very high and in most cases exceeded 70% of the total cell number (Fig. 2, left panel).

In the presence of prophage, the amount of elongated cells increased more than 2-folds for lambda and 2.5-folds for the 32Δtox and 933WΔtox prophage in the first hour of addition of mitomycin C, when compared to a strain without a prophage. Further into experiments, the difference in amount of elongated cells between prophage-free and prophage containing strains was not so great but still it was higher for strains with a prophage than for MG1655 without a prophage. Under growth conditions where no inducing agents were added, the amount of filamenting cells obtained for strains with tested prophages was similar to the control strain without a prophage (Fig. 2, left panel). There is no clear correlation for the phage titer and the amount of elongated cells formed.

### Discussion

Even though *Escherichia coli* belongs to the human normal gut flora, it can be also very harmful. In fact, there are several strains of *E. coli* which show pathogenicity against humans. In the case of *E. coli* O157:H7, the main virulence factor is the Shiga toxin that inhibits translation in eukaryotic cells. Production and release of the Shiga toxin depend on induction of prophages which encode this toxin among their late genes. Prophage induction can result from the action of the human immune system. Presence of human neutrophils in an EHEC culture has led to production and release of the Shiga toxin (Wagner et al. 2001). Neutrophils take part in the immune response which may happen after EHEC infection and use hydrogen peroxide to kill pathogens (Liu et al. 1999), and in turn, Shiga toxin-producing bacteriophages are induced under thus caused oxidative stress conditions (Łoś et al. 2009, 2010).

The results presented here show that sensitivity of bacterial cells and their reaction to hydrogen peroxide depend to a great extent on the type of prophage carried. When cells are more sensitive to oxidative stress and trigger the SOS response, more Shiga toxin can be produced and released, but also, potential differences in induction sensitivity of prophage itself may play a key role here. This can be one of the potential explanations as to why different EHEC strains produced different amounts of the Shiga toxin (Wagner et al. 1999).

In the case of phage induction with mitomycin C in this study, there is no significant difference in sensitivity of bacterial cells belonging to the same strain but lysogenized with different prophages. However, presence of the prophage increases the amount of cells reacting by filamentation in response to mitomycin C presence, especially in the first hour after addition of this agent.

The results of presented experiments show that many more *E. coli* cells react by filamentation in response to mitomycin C than to hydrogen peroxide. That may implicate a deeper adaptation, as hydrogen peroxide is used by neutrophils, but it is also used by, e.g., the eukaryotic predator *Tetrahymena thermophila*. EHEC strains produce and release Shiga toxin in the presence of *T. thermophila* and kill this protozoa (Lainhart et al. 2009). Induction of prophages during oxidative stress can be used by the host as a defense mechanism against predators, by inducing a fraction of bacterial population, which will release Shiga toxin to protect the remaining cells, creating a sort of a community defense system (Łoś et al. 2013). However, it was shown that it is not effective in the case of all grazing protozoa (Schmidt et al. 2016).

The results presented in this study indicate that in most lysogenic strains, about 20 to 35% of cells react by filamentation within 2 h after addition of hydrogen peroxide. This fraction of the population may provide defense against the immune system or a predator after release of the Shiga toxin, while the remaining, not filamenting bacterial cells may overcome the oxidative stress and survive without triggering prophage induction. This may suggest a random reaction based on the physiological state of the cell, but also may indicate more complicated interactions, where the defender fraction of cells in a population sacrifices itself to protect presisters on the basis of some signals which may resemble quorum sensing mechanisms. In the case of the 27Δtox and 32Δtox prophages, 66% and 70% of cells, respectively, are elongated after 1 h of the experiment. Despite this fact, bacterial culture growth was still observed and there was no drop in OD measurements during the course of the experiment, when disappearance of elongated cells suggested phage-driven lysis and thus killing of the cells contributing significantly to the OD readouts and additionally at certain point in time constituting a significant fraction of the cells in the culture.

In the case of O57:H7 EDL933 which is a clinical isolate, the amount of elongated cells was higher and persisted for the duration of the whole experiment. A total of 30 to 40% of bacterial cells reacted to the addition of hydrogen peroxide by filamentation. This may reflect a better adaptation of this strain to co-operate with the prophage, as it seems to be more effectively induced, and thus in case of an attack by hydrogen peroxide, the Shiga toxin is produced and released more efficiently by a bacterial community, as long as the danger, like a predator or a neutrophil, is present. The reason why only part of the bacterial culture reacts to the stress conditions could be due to population heterogeneity in phage induction that interferes with the extinction of the lysogen population. This heterogeneity can be modulated by environmental conditions (Imamovic et al. 2016).

It is interesting that only a few serotypes of *E. coli* are linked with this potentially deadly infection, while phages carrying the *stx* genes are known to infect other *E. coli* types (Iversen et al. 2015). In our study, we showed that the pattern of phage induction by H_2_O_2_ is dependent on the prophage itself, but even more, it depends on the bacterial strain. In this study, H_2_O_2_ had proven to be a relatively mild inducer of prophages present in the MG1655 strain, when compared to mitomycin C. Hydrogen peroxide had induced a transient slowdown of bacterial culture growth and a sharp peak in cell filamentation. In the case of EDL933, the same concentration of hydrogen peroxide gave a much more dramatic effect, with the prophage induction resulting in a phage titer that was very close to the one obtained by using mitomycin C. It also resulted in prolonged slowdown of growth and much higher, and relatively stable over time, cell filamentation in the culture. Also, in the case of EDL933, a better correlation between the observed maximum amount of elongated bacterial cells and the final phage titer was observed than for the MG1655-based lysogens, where relatively high values of the elongated bacteria after hydrogen peroxide induction did not correspond to the final phage titers, when compared to the mitomycin C induction. This may suggest that the process of the phage induction by hydrogen peroxide is strongly dependent on the strain itself, and this in turn may predispose some *E. coli* bacterial strains to become effective pathogens when paired with a proper phage carrying the Shiga toxin.

Our results also demonstrated the limitation of the use of model organisms in order to understand some mechanisms and to extrapolate the results into real-life situations. In the case of *E. coli* EDL933, mitomycin C is a very strong prophage inducer and it causes similar effects, including growth inhibition followed by culture lysis. This could also be observed in the case of lysogenic MG1655, where the reaction to hydrogen peroxide addition was much more intense, when compared to the model MG1655 strain. In the case of EDL933, the induction caused by hydrogen peroxide was much stronger and more stable in terms of both: phage titer and cell filamentation. It seems that there was a good correlation between cell filamentation and phage production by the culture, but the number of phages produced is not directly proportional to the observed fraction of filamenting cells; instead, effectiveness of phage production rose dramatically when mitomycin C was used (even by a few orders of magnitude), when compared to induction by hydrogen peroxide, with a maximum of 2–3-fold differences observed. This difference may be at least partially due to a much shorter half-life of hydrogen peroxide in the culture, due to effective inactivation, when compared to mitomycin C, which is mostly resisted by *E. coli* cells by using DNA repair, and not inactivation or effective removal of the antibiotic (Bolt et al. 2016).
